# Differences in predictions of ODE models of tumor growth: a cautionary example

**DOI:** 10.1186/s12885-016-2164-x

**Published:** 2016-02-26

**Authors:** Hope Murphy, Hana Jaafari, Hana M. Dobrovolny

**Affiliations:** Department of Physics, Utica College, Utica, NY USA; Department of Physics & Astronomy, Texas Christian University, 2800 S. University Drive, TX, 76129, Fort Worth USA

**Keywords:** Tumor growth, Mathematical model, Ordinary differential equation, Cancer, Chemotherapy

## Abstract

**Background:**

While mathematical models are often used to predict progression of cancer and treatment outcomes, there is still uncertainty over how to best model tumor growth. Seven ordinary differential equation (ODE) models of tumor growth (exponential, Mendelsohn, logistic, linear, surface, Gompertz, and Bertalanffy) have been proposed, but there is no clear guidance on how to choose the most appropriate model for a particular cancer.

**Methods:**

We examined all seven of the previously proposed ODE models in the presence and absence of chemotherapy. We derived equations for the maximum tumor size, doubling time, and the minimum amount of chemotherapy needed to suppress the tumor and used a sample data set to compare how these quantities differ based on choice of growth model.

**Results:**

We find that there is a 12-fold difference in predicting doubling times and a 6-fold difference in the predicted amount of chemotherapy needed for suppression depending on which growth model was used.

**Conclusion:**

Our results highlight the need for careful consideration of model assumptions when developing mathematical models for use in cancer treatment planning.

## Background

Cancer is a leading cause of death and places a heavy burden on the health care system due to the chronic nature of the disease and the side effects caused by many of the treatments [1–3]. Much research effort is spent improving the efficacy of current treatments [4] and on developing new treatment modalitites [5–9]. As cancer treatment moves towards personalized treatment, mathematical models will be important component of this research, helping to predict the time course of the tumor and optimizing treatment regimens [10, 11].

Mathematical models are used in a number of ways to help understand and treat cancer. Models are used to understand how cancer develops [12] and grows [13–16]. They are used to optimize [17, 18] or even personalize [11, 19, 20] current treatment regimens; predict the efficacy of new treatments [21] or combinations of different therapies [22–24]; and give insight into the development of resistance to treatment [25, 26]. While models have great potential to improve development and implementation of cancer treatment, they will only realize this potential if they provide accurate predictions.

The basis of any mathematical model used to study treatment of cancer is a model of tumor growth. This paper focuses on ordinary differential equation (ODE) models of tumor growth. A number of ODE models have been proposed to represent tumor growth [27, 28] and are regularly used to make predictions about the efficacy of cancer treatments [29]. Unfortunately, choice of a growth model is often driven by ease of mathematical analysis rather than whether it provides the best model for growth of a tumor [27].

Some researchers have attempted to find the “best” ODE growth model by fitting various models to a small number of experimental data sets of tumor growth [30–33]. Taken altogether, the results are rather inconclusive, with results suggesting that choice of growth model depends at least in part on the type of tumor [31, 32]. This leaves modelers with little guidance in choosing a tumor growth model.

Many researchers realize that improper choice of growth model is problematic [27] and can lead to differences in predictions of treatment outcomes [28, 29]. However, there has not yet been a study that compares and quantifies differences in predictions of the various models and how these differences affect predictions of treatment outcomes. This paper presents results of analysis of the various ODE growth models highlighting their predictions of tumor growth in the presence and absence of chemotherapy. We also fit the models to sample experimental tumor growth data sets and find a wide range of predicted outcomes based on the choice of growth model.

## Methods

### Mathematical models

Early studies of tumor growth were concerned with finding equations to describe the growth of cancer cells [13–16] and many of the models examined here were proposed at that time. The models predict the growth of a tumor by describing the change in tumor volume, *V*, over time. The model equations used in this analysis are presented in Table 1 and the models are described below. *a*, *b*, and *c* are parameters that can be adjusted to describe a particular data set.

**Table 1:** ODE models of tumor growth

Model	Equation
Exponential	${\dot {V}=a V}$
Mendelsohn	${\dot {V}=a V^{b}}$
Logistic	${\dot {V}=a V \left (1-\frac {V}{b}\right)}$
Linear	${\dot {V}= \frac {aV}{(V+b)}}$
Surface	${\dot {V}= \frac {aV}{(V+b)^{\frac {1}{3}}}}$
Gompertz	${\dot {V}=aV\ln {\frac {b}{(V+c)}}}$
Bertalanffy	${\dot {V}=aV^{\frac {2}{3}}-bV}$

**Exponential:** In the early stages of tumor growth, cells divide regularly, creating two daughter cells each time. A natural description of the early stages of cancer growth is thus the exponential model [34], where growth is proportional to the population. The proportionality constant *a* is the growth rate of the tumor. This model was often used in early analysis of tumor growth curves [13–16] and appears to work quite well at predicting early growth. It is known to fail, however, at later stages when angiogenesis and nutrient depletion begin to play a role [27, 32].

**Mendelsohn:** A generalization of the exponential growth model was introduced by Mendelsohn [35]. In this model, growth is proportional to some power, *b*, of the population.

**Logistic:** The logistic (or Pearl-Verhulst) equation was created by Pierre Francois Verhulst in 1838 [36]. This model describes the growth of a population that is limited by a carrying capacity of *b*. The logistic equation assumes that the growth rate decreases linearly with size until it equals zero at the carrying capacity.

**Linear:** The linear model assumes initial exponential growth that changes to growth that is constant over time. In our formulation of the model, the initial exponential growth rate is given by *a*/*b* and the later constant growth is *a*. The model was used in early research to analyze growth of cancer cell colonies [16].

**Surface:** The surface model assumes only a thin layer of cells at the surface of the tumor are dividing while the cells inside the solid tumors do not reproduce; they are mitotically inactive [37]. Our formulation again assumes exponential growth at early times with the surface growth taking over at longer times.

**Bertalanffy:** The Bertalanffy equation was created by Ludwig Bertalanffy as a model for organism growth [38]. This model assumes that growth occurs proportional to surface area, but that there is also a decrease of tumor volume due to cell death. This model was shown to provide the best description of human tumor growth [30].

**Gompertz:** Benjamin Gompertz originally created the Gompertz model in 1825 in order to explain human mortality curves [39]. The model is a generalization of the logistic model with a sigmoidal curve that is asymmetrical with the point of inflection. The curve was eventually applied to model growth in size of entire organisms [40] and more recently, was shown to provide the best fits for breast and lung cancer growth [32].

### Dynamical analysis

Our goal is to assess differences in model predictions. While we are often concerned with prediction of time points in the near future, it is also informative to study the long-term predictions of a mathematical model. To this end, we find the fixed points of each equation which will tell us the long-term predictions of each of the models. Stability analysis [41] is used to determine the boundary between growth and decay of the tumor.

We also determine the doubling time,

(1) $$ DT=\frac{\text{ln}2}{\lambda},   $$

where *λ* is the initial growth rate of the tumor. The doubling time is often used as a measure of how fast the tumor grows [42]. We use a Taylor expansion of the equations in Table 1 about *V*=0 to determine the initial growth rate. While this means that the calculated doubling time is an approximation and only valid during the early portion of the growth phase, many experimental data sets only follow the growth for a short period of time so this is representative of what might be calculated in actual experiments.

### Chemotherapy

In addition to assessing the predictions of the growth models alone, we examined how predictions differed when chemotherapy was added to the models. This is particularly important since growth models are often used as a basis for predicting the efficacy of cancer therapies.

Since this is just illustrative, we choose a simple implementation of chemotherapy. We assume that there is a constant supply of drug *C*_0_ acting on the tumor. We simply subtract the term *C*_0_*V* from each equation [29] and again use stability analysis to determine the conditions that lead to eradication of the tumor.

### Data fitting

Data from Worschech et al. [43] of a GI-101A xenograft in nude mice (Figure 1A of [43], control data) was extracted using WebPlotDigitizer, an online data extraction tool. Fitting was performed by minimizing the sum of squared residuals (SSR),

(2) $$ \text{SSR}= \sum_{i} (x_{i} - m_{i})^{2},  $$

where *x*_*i*_ are the experimental data points, and *m*_*i*_ are the predicted model values at the same times. The lowest SSR was found using the Python Scipy fmin_tnc function, which uses a truncated Newton algorithm.

Since the models have a different number of free parameters, comparison using only the SSR is not always fair since models with more free parameters have more freedom to to fit the data. To correct for this bias, we use Aikaike’s information criterion (AIC_*C*_), corrected for small sample size, which penalizes models with more parameters if there is not enough improvement in the SSR. The AIC_*C*_ is given by

(3) $$ \text{AIC}_{C} = n\text{ln}\left(\frac{SSR}{n}\right)+\frac{2(K+1)n}{n-K-2} \,   $$

where *n* is the number of data points and *K* is the number of parameters [44]. The model with the lowest AIC_*C*_ is considered to be the better model given the experimental data it is approximating.

## Results

### Tumor growth in the absence of chemotherapy

A simple analysis of the different models shows that they have very different predictions of the long-term dynamics of tumor growth. The fixed points, doubling time and condition for growth of the tumor are presented in Table 2. All models have two fixed points, one of which is zero. The remaining fixed point represents the maximum possible tumor size predicted by the model. In a real system, the maximum possible tumor size, or carrying capacity, is a function of the tumor’s environment and its access to resources [45] and can change as the tumor grows, particularly in the case of extracapsular extension when it extends beyond the bounds of its original organ. Four of the models (exponential, Mendelsohn, linear, and surface) predict that tumors will continue growing without bound, a biologically unrealistic scenario. The remaining three models (logistic, Gompertz, and Bertalanffy) predict that tumors will grow to some maximum size and reach a stable equilibrium at that point.

**Table 2:** Model predictions in the absence of chemotherapy

Model	Maximum	Doubling	Growth
	size	time	condition
Exponential	*∞*	$\frac {\ln 2}{a}$	*a*>0
Mendelsohn	*∞*	$\frac {\ln 2}{a}$	*a*>0
Logistic	*b*	$\frac {\ln 2}{a}$	*a*>0
Linear	*∞*	$\frac {b\ln 2}{a}$	$\frac {a}{b}>0$
Surface	*∞*	$\frac {b^{\frac {1}{3}}\ln 2}{a}$	$\frac {a}{b^{\frac {1}{3}}}>0$
Gompertz	*b*−*c*	$\frac {\ln 2}{a\ln \left (\frac {b}{c}\right)}$	$a\ln \left ({\frac {b}{c}}\right)>0$
Bertalanffy	$\left (\frac {a}{b}\right)^{3}$	${\frac {\ln 2}{a-b}}$	*a*−*b*>0

The growth criteria listed in Table 2 gives the condition for growth or decay of the tumor if a few cancer cells appear in the system. While the criteria all have slightly different forms, they essentially tell us that the initial growth rate once tumor cells appear must be positive. All the models agree that if the initial growth rate is positive, the tumor will continue to grow until it reaches its maximum size; the disease-free equilibrium is unstable. The doubling time for each model gives an indication of how quickly the tumor will reach this maximum size. Unfortunately, comparing the formulas does not really give much insight into differences in model predictions without having some estimate of the parameter values. In a later section, we give a quantitative assessment of differences in model predictions using sample tumor growth data.

### Tumor growth in the presence of chemotherapy

As described in Methods, we assess how chemotherapy alters the dynamics of each of the growth models using the simplifying assumption of constant drug concentration. We again use stability analysis to assess the long-term predictions made by each of the models. Each of the models again predicts that there are two possible fixed points, one of which is zero. The other fixed point represents the maximum possible tumor size in the presence of chemotherapy and is presented in Table 3. In this case, only one model (exponential) predicts that the tumor will continue to grow indefinitely even in the presence of chemotherapy. The remaining models all predict that the chemotherapy will hold the tumor to some maximum size. Unfortunately, it is again difficult to assess the relative sizes of the predicted maximum size without having values for parameters.

**Table 3:** Model predictions in the presence of chemotherapy

Model	Maximum	Minimum concentration
	size	needed to cure
Exponential	*∞*	*C*_0_=*a*
Mendelsohn	$\left (\frac {C_{0}}{a}\right)^{\frac {1}{b-1}}$	*C*_0_=*a*
Logistic	$\frac {b(a - C_{0})}{a}$	*C*_0_=*a*
Linear	$\frac {a}{C_{0}} - b$	$C_{0}= \frac {a}{b}$
Surface	$\frac {a^{3}}{C_{0}} - b$	$C_{0}=\frac {a}{b^{\frac {1}{3}}}$
Gompertz	$\frac {b}{e^{\frac {C_{0}}{a}}} - c$	$C_{0}=a\ln \left (\frac {b}{c}\right)$
Bertalanffy	$\left (\frac {a}{b+C_{0}}\right)^{3}$	*C*_0_=*a*−*b*

We can again determine the boundary condition that delineates growth of the tumor from decay of the tumor. In this case, this represents the minimum amount of chemotherapy needed to cause eradication of the tumor. Essentially, the minimum amount of chemotherapy needed is the amount that results in a kill rate equal to the initial growth rate of the tumor.

### Quantitative example

In the previous sections, we derived equations for maximum tumor size and conditions for growth of the tumor in the presence and absence of chemotherapy for each of the ODE growth models. However, it is difficult to assess just how large differences between model predictions are without having values for model parameters. In this section, we use sample tumor growth data extracted from the literature to quantitatively assess differences in model predictions.

We use data from Worschech et al. [43] which consists of measurements of growth of GI-101A cells injected subcutaneously into nude mice. This is an unusually long data set consisting of 14 time points spanning 114 days. In addition to assessing differences in model predictions, we will use this data set to examine whether model predictions can be improved with the collection of more data. We will initially use only the first half of the time series, seven points spanning 65 days. Note that many tumor growth data sets contain fewer than ten points and often span only a week or two [31], so this truncated data set is quite representative of much of the data available in the literature.

**Fig. 1:**
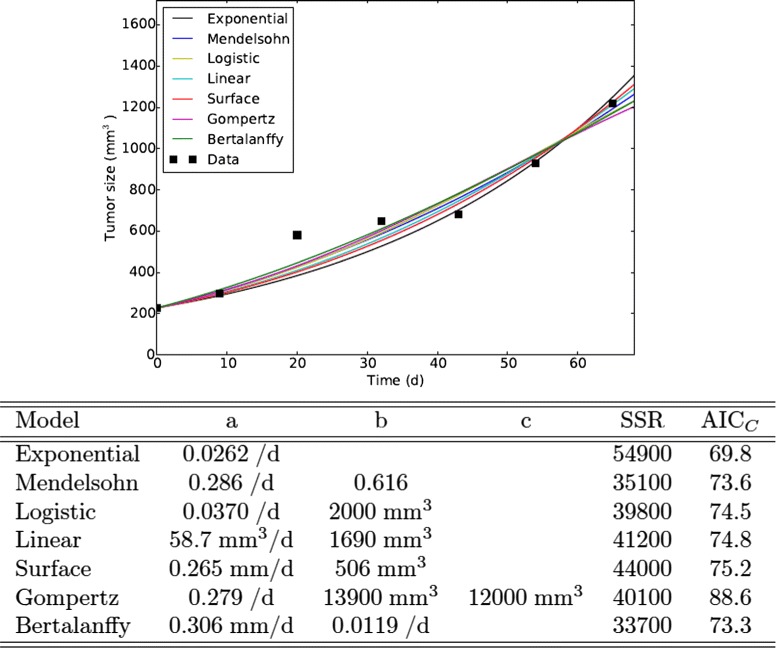
Model fits to data. Best fits of the ODE tumor growth models to the first half of the data from Worschech et al. [43]. Parameter estimates are given in the table below the graph

Model fits to this truncated data, along with the best fit parameter estimates are presented in Fig. 1. All the models provide reasonable fits to the data, with the exponential model producing the worst SSR since it only has one free parameter. The model with the lowest SSR is the Bertalanffy model in this case. However, the AIC_*C*_ indicates that the exponential model actually provides the best explanation for the data since the improvement in SSR did not offset the inherent improvement in fit with the addition of the extra parameter. A close inspection of the fits shows that they largely agree on the growth trajectory while there are experimental data points to guide the time course, but they appear to diverge beyond the last experimentally collected time point. This is particularly problematic since mathematical models are often used for extrapolation, suggesting that proper choice of growth model is extremely important for correctly predicting the future growth of tumors as well as for assessing how treatment might affect growth of the tumor.

**Fig. 2:**
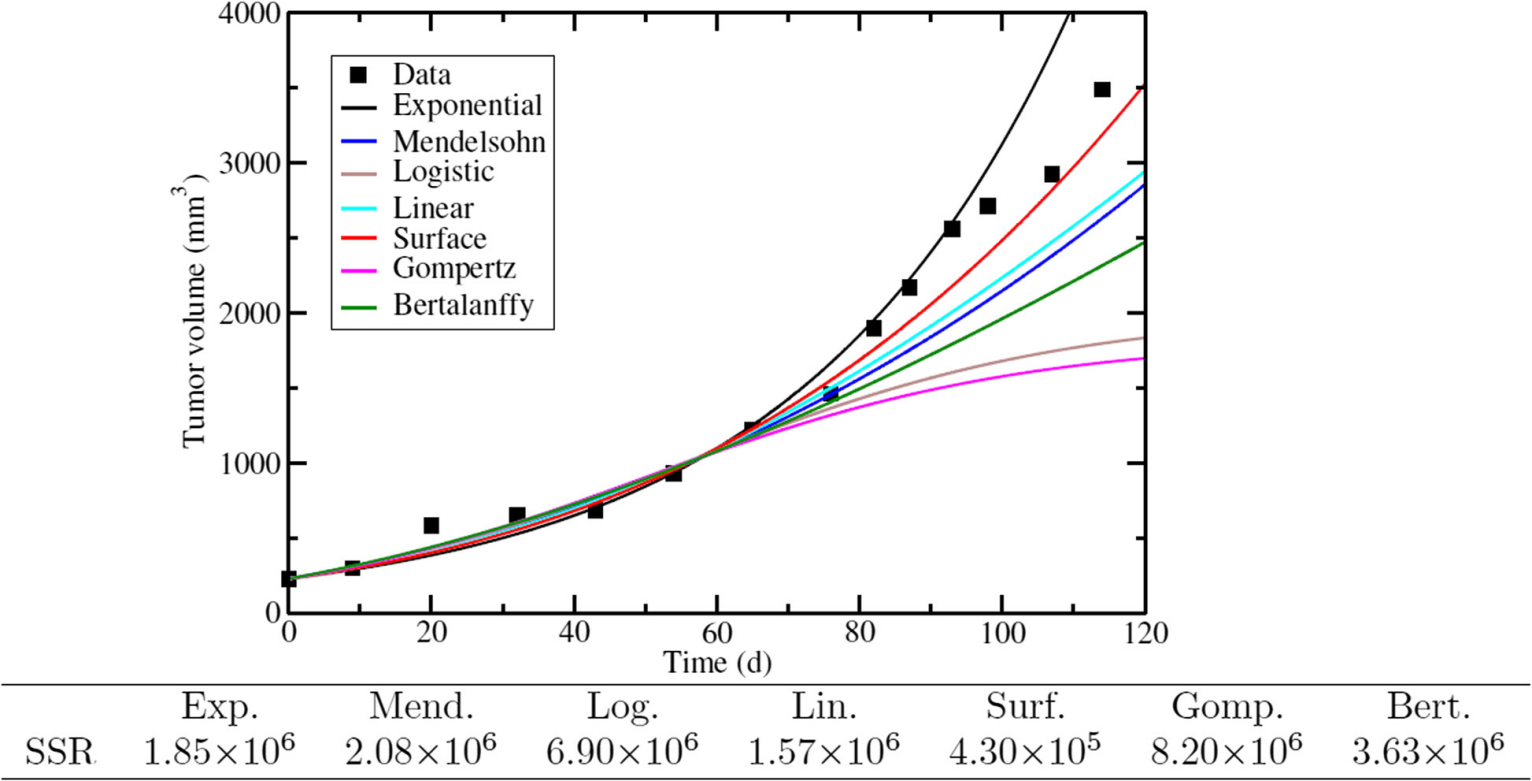
ODE models’ predicted time course of tumor growth. Each model was fit to the first seven time points and parameter estimates were used to extrapolate the remaining seven time points. The SSR for each prediction is given in the table below the graph

As a test of the accuracy of each model, we can use the best fit parameter estimates from the truncated data to predict the remaining seven time points of the full data set. As a measure of the accuracy of the predictions, we can calculate the SSR for each model prediction. The model predictions, along with the SSRs, are presented in Fig. 2. While the model that provided the best fit to the data was the Bertalanffy model and the model that provided the best explanation for the data was the exponential model, the model that actually provides the best estimate of the future growth of the tumor is the surface model. This is likely because the experimental data are measurements of a xenograft which grows as an approximately spherical tumor where only the cells near the surface are dividing. With the exception of the exponential model, the models underestimate the actual growth of the tumor. In the case of the Bertalanffy, Gompertz, and logistic models, this is because the truncated data set did not provide enough information to correctly estimate the maximum tumor size. Unfortunately, these three models are particularly popular choices for modeling tumor growth [27, 29] because they include a biologically realistic slowing of the growth rate as the tumor increases. Yet it is precisely this feature that results in the poor predictive value of the models.

In practice, mathematical models are often not used to predict full time series, but are used to calculate quantities of interest to clinicians. Using the formulas derived in sections “Tumor growth in the absence of chemotherapy” and “Tumor growth in the presence of chemotherapy”, we can use our parameter estimates to calculate maximum tumor size, doubling time, and minimum concentration of chemotherapy needed for suppression of the tumor. These quantities are presented in Fig. 4 (top row) for the truncated Worschech data. Four of the models (exponential, Mendelsohn, linear, and surface) predict indefinite growth of the tumor. The remaining three models predict finite tumor sizes, but the predicted maximum size varies by almost an order of magnitude, with the Gompertz and logistic models estimating a maximum tumor volume of ∼2000mm^3^ while the Bertalanffy model estimates a maximum tumor volume of ∼16000mm^3^. The doubling time estimated by the different models also shows a good deal of variation, ranging from ∼2 d for the Mendelsohn and Bertalanffy models to ∼26 d for the exponential model. The assumption of exponential growth underlies many calculations of the tumor growth rate or doubling time [42, 46] and the exponential model is also the model of choice for this data, so it is concerning that the exponential model provides one of the extreme estimates of doubling time. Of particular concern is the variation in predictions of the minimum amount of chemotherapy needed to suppress a tumor. The Bertalanffy and Mendelsohn models predictions are about six times larger than the predictions of the remaining models. If we use one of these models to decide on treatment plans, we could be treating patients with far more drug than is actually necessary. The extreme values predicted by the Bertalanffy model are especially concerning since the Bertalanffy model provided the lowest SSR and might be a choice for some modelers in predicting the future growth of this particular tumor.

**Fig. 3:**
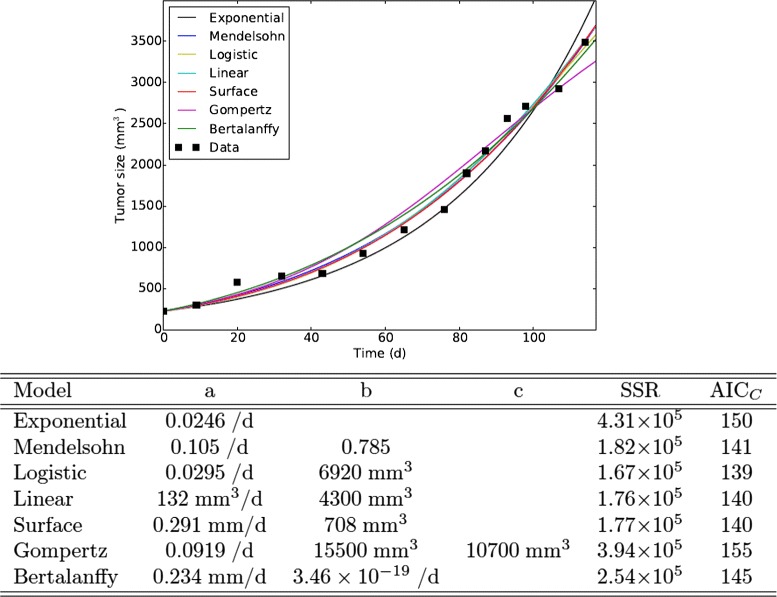
Model fits to data. Best fits of the ODE tumor growth models to the data from Worschech et al. [43]. Parameter estimates are given in the table below the graph

Given that the short time series led to a large variation in predicted outcomes, we examined whether the collection of extra time points might lead the models to more closely agree on predicted outcomes. We fit the full Worschech time series with each of the ODE growth models, as shown in Fig. 3. Many of the estimated parameter values change somewhat from the estimates determined by the fits to the first half of the time series. The most notable of these is the second parameter (*b*) of the Bertalanffy model which drops to essentially zero, suggesting that the best description of the data by this model neglects death within the core of the tumor. The model with the best fit in this case is the logistic model, which has both the lowest SSR and lowest AIC_*C*_, so the addition of extra information can alter the choice of growth model. Again, however, we see that the models all provide reasonably good fits to the experimental data, but start to diverge beyond the last data point. It is unclear if this divergence will lead to large variations in clinical parameters.

**Fig. 4:**
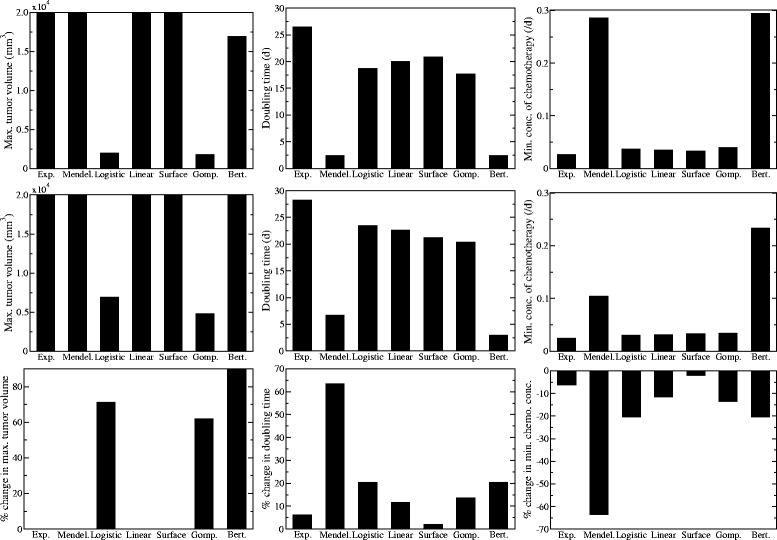
Estimates of clinically important measurements. Model predictions of the maximum tumor volume (*left*), doubling time (*center*), and minimum concentration of chemotherapy needed for eradication (*right*) based on parameter estimates from the half (*top row*) or the full (*center row*) Worschech data set. The bottom row shows the percent change in each of the predictions when the full data set is used rather than the truncated data set

The maximum tumor size, doubling time and minimum amount of chemotherapy needed for suppression predicted by each model based on parameter estimates from the full Worschech time series are shown in Fig. 4 (center row). As before, four of the models predict unfettered growth of the tumor, but they are now joined by the Bertalanffy model in predicting unrealistically large tumors. Since there is now essentially no death of tumor cells in the Bertalanffy model, the tumor continues to grow indefinitely. The maximum tumor sizes predicted by the Gompertz and logistic models have increased slightly to ∼5000 mm^3^ and ∼7000 mm^3^, respectively. This is because the new data clearly shows that the tumor does not stop growing at 2000 mm^3^. The doubling times predicted by the Mendelsohn and Bertalanffy models are still quite a bit smaller than those predicted by the remaining models, although these estimates have increased. Finally, the predicted amount of chemotherapy needed to suppress the tumor by the Mendelsohn model drops, coming noticeably closer to the values predicted by all but the Bertalanffy model.

To quantify the changes we see with the addition of extra time points, we calculate the percent difference in each prediction between estimates based on the truncated time series and estimates based on the full time series (Fig. 4, bottom row). Of those models that predict a finite tumor size, we see that all have increased the predicted size of the tumor. The predicted doubling time has also increased for all of the models. This suggests that all of the models were underestimating the true doubling time of the tumor. Similarly, the percent differences suggest that the models all overestimated the amount of chemotherapy needed to suppress the tumor. The Mendelsohn and Bertalanffy models, which predicted particularly small doubling times and large amount of chemotherapy, show the largest percent changes in both estimates with the addition of extra time points. The surface model, which most accurately predicted the full time course based on estimates from the first half, shows the smallest percent change with the addition of extra time points.

## Discussion

This paper examines several commonly used ODE models of tumor growth and quantitatively assesses the differences in their predictions of clinically relevant quantities. We first derived equations for the maximum tumor size, doubling time, and the condition for growth of all the models. We then derived equations for the maximum tumor size in the presence of chemotherapy and the minimum amount of chemotherapy needed to suppress a tumor. Finally, we used experimental tumor growth data along with these equations to compare predicted values of maximum tumor size, doubling time, and minimum amount of chemotherapy needed for suppression for each of the ODE models. We find that there is a six-fold difference in the minimum concentration of chemotherapy required for suppression of the tumor and a 12-fold difference in estimates of the doubling time. While the exact amount of variation in predictions between different models will differ for other data sets, we expect that there will be disagreement in model predictions for all data sets. In fact, this data set was particularly long, so the models were constrained to agree for a longer time period than with most other data sets. This, along with our finding that increasing the duration of the data set reduced the variability in model predictions suggests that differences in model predictions might be even larger for most other data sets. These findings suggest that modelers and clinicians must carefully consider their choice of growth model and how different growth assumptions might alter model predictions of the efficacy of treatment.

While our findings could be dismissed because they are based on a single example or because the models and the implementation of chemotherapy are highly simplified, we believe they highlight a significant problem. While many mathematical models used for clinical assessment of patients and development of radiation or chemotherapy plans are more complex than those presented here [47], they must all make some assumption of how the tumor will grow. Due to the complexity of these models, however, it is difficult to trace the effect of the choice of growth model and determine how this choice might alter the model’s predictions. In fact, while model predictions are often assessed for sensitivity to errors in estimates of the parameters [48, 49], the effect of model assumptions is often neglected. Our findings, however, indicate that these assumptions could have a profound effect on model predictions since our simple models show that different choices of growth model result in large variations in model predictions. The results of these inaccuracies could have significant impacts on patient outcomes since we might either provide too much treatment, causing more severe side effects, or too little treatment, possibly resulting in continued growth of the tumor. In fact, a recent analysis of patients receiving radiation therapy suggests that tumor size relative to its maximum possible size is a stronger indicator of response to treatment than simply the tumor size [50]. This is because the radiosensitivity of tumor cells is dependent on their growth and tumors closer to their maximum size are growing more slowly than tumors that still have room to grow. This simply highlights the need to accurately determine how tumors are growing when planning for dose and fractionation schedule.

While some research has attempted to find the best ODE model to describe tumor growth [30–33], the results seem to suggest that there are no broad guidelines; the most appropriate model seems to be dependent on the details of the experiment. These papers used least-square minimization, or minimization of information criterion to determine the “best” model [44]. In our example, use of minimum SSR would lead us to choose the Bertalanffy model as the “best” model, while use of AIC_*C*_ would lead us to choose the exponential model to fit the truncated Worschech data set. However, further investigation suggests that either of these models would actually be a poor choice of model. The Bertalanffy did a poor job of predicting the future growth of the tumor (Fig. 2), and gave an extremely low estimate of the doubling time and a high estimate for the amount of chemotherapy needed to suppress the tumor. The exponential model overestimated the growth rate of the tumor and does not allow for slower growth of the tumor as resources are depleted.

While some modelers would perhaps fit several different growth models to a data set, current model selection techniques were not designed for the type of model selection problem faced by cancer modelers. Statistical measures such as the SSR, AIC_*C*_, Mallow’s *C*_*p*_ [51], Schwarz Bayesian information criterion [52], among others, all measure how well the model explains experimental data that has already been collected. A model selected as the best model using one of these measures should work reasonably well to make predictions if future behavior is similar to past behavior. Unfortunately, we know that this is often not the case when modeling tumor growth. Most experimental data sets capture the early growth of the tumor [31]. Modelers, however, would like to predict future growth where space and resource limitations hamper growth and structural changes such as a necrotic core, extracapsular extension, and angiogenesis will also affect growth dynamics [53–55], so the data used to select the model does not necessarily reflect the dynamics at the time when the predictions are made. In addition, it is well-known that experimental results in many pre-clinical systems do not translate well to human clinical studies [56–59]. A model chosen based on goodness-of-fit criteria to data from a pre-clinical experiment might not provide the most accurate predictions of future growth and treatment outcomes in humans. Our example suggests that more robust testing of model assumptions is needed before settling on a particular formulation. Minimization of SSR or information criterion does not guarantee selection of the best model for predicting future behavior.

## Conclusions

Our results show that choice of tumor growth model can lead to as much as a 12-fold change in predicted outcomes and that the model that best fits experimental data might not be the model that best predicts future growth. It is our hope that the findings presented here will spur more investigation into the effect of choice of cancer growth model on predicted treatment outcomes and that researchers will consider more than just best fit when selecting a growth model.

## Abbreviations

ODE: ordinary differential equation
SSR: sum of squared residuals
AIC_*C*_: aikaike’s information criterion
